# The Relationship between Parenting Styles and Adolescents’ Social Anxiety in Migrant Families: A Study in Guangdong, China

**DOI:** 10.3389/fpsyg.2017.00626

**Published:** 2017-04-20

**Authors:** Jihong Xu, Shiguang Ni, Maosheng Ran, Chengping Zhang

**Affiliations:** ^1^National Research Institute for Family PlanningBeijing, China; ^2^Graduate School at Shenzhen, Tsinghua UniversityShenzhen, China; ^3^Department of Social Work and Social AdministrationThe University of Hong Kong, Hong Kong, Hong Kong

**Keywords:** parenting styles, social anxiety, adolescents, migrant families, emotional warmth, overprotection, rejection

## Abstract

Previous studies indicated that parenting styles were important influencing factors for the development of children’s well-being. It is known that mass migration to the cities in China will affect family relations. However, few studies focused on the relationship between parenting styles and adolescents’ mental health in migrant families. Thus, this study aimed to investigate how parenting styles could affect adolescent’s social anxiety in migrant families. A total number of 1,345 adolescents in migrant families from four non-government-funded junior middle schools in Guangdong province formed the research sample. Parenting styles were measured using short-form of the Egna Minnen Beträffande Uppfostran, and social anxiety was evaluated using Social Anxiety Subscale of Self-Consciousness Scale. The results showed that emotional warmth, overprotection and rejection were significantly more often perceived from mothers than from fathers. Significant group differences between high social anxiety group and low social anxiety group were found in both father’s rearing styles and mother’s rearing styles. Furthermore, in migrant families, paternal emotional warmth could decrease adolescents’ social anxiety, whereas maternal overprotection could increase it.

## Introduction

As economy and the urbanization rate grow rapidly in China, more and more rural surplus labors left countryside and flocked to big cities, and the floating population has been skyrocketing. In recent years, family migration has become the main mode of China’s floating population migration, which means that more and more parents took their children out of their registered permanent residence to the new cities where they worked and lived. The number of adolescents in migrant families has increased dramatically during the past years, reaching 35.81 million by the end of 2010 (All-China women’s federation, 2013). During this immigrating process, children and adolescents in migrant families often have to face great pressure in order to adapt to the life in cities, which gives rise to various mental health problems. Evidence showed that the incidence rate of children’s mental health problems in migrant families was higher than that in non-migrant families (Xia, 2015). Lin et al. (2009) found that children in migrant families had significantly more psychological problems, such as loneliness, depression, and social anxiety than local children.

Social anxiety is the main psychological problem for children and adolescents, and it not only affects individual’s daily communication, but also influences their mental health. Social anxiety is defined as a common human experience characteristic that is triggered by an intense fear of evaluation from others in social interactions (Morrison and Heimerg, 2013), and serious social anxiety is likely to develop into social anxiety disorder. The prevalence of social anxiety peaks during the early to middle adolescent years (Steinberg and Morris, 2001). A few studies in China showed that adolescents had become the high-risk group of social anxiety (Peng, 2004), and the incidence rate of social anxiety disorder for adolescents aged between 13 and 24 reached 8%. Besides the genetic factors (Hettema et al., 2005), the factors such as information processing, parental rearing and modeling, family functioning can also influence the children’s social anxiety (e.g., Bögels et al., 2011). In addition, mounting studies investigated the relationship between children’s social anxiety and parental rearing (Bögels et al., 2011; Mothander and Wang, 2014).

Parenting style is one of the important factors in family education and is a relatively stable behavior pattern and tendency in raising and educating children through daily activities. Parenting styles generally fall along a continuum between the two anchors of being lax and overly punitive, with extremes in either direction defined as negative (Stevens, 2014). Positive parenting is a strategy that involves warmth, sensitivity, acceptance and responsiveness toward the child (Kawabata et al., 2011). Previous studies indicated that parenting style and idea were important influencing factors for children’s psychological and behavioral development (Brennan et al., 2013). In the studies of children’s parenting style in Chinese migrant families, parents tended to usually adopt some negative parenting styles (e.g., punishment and authoritarian) and seldomly adopt positive parenting styles (e.g., emotional warmth and understanding) (Ma et al., 2015). Zhang and Li (2011) and Verhoeven et al. (2012) reported that poor parenting style would increase the incidence rate of children’s psychological problems, and the negative parenting styles, such as overprotection and excessive interference, were more likely to increase children’s social anxiety. Other studies had also shown that rejective (Brown and Whiteside, 2008), overprotective (Bögels et al., 2001), and anxious (Gruner et al., 1999; Muris et al., 2000) parenting styles were related to the increase of children’s anxiety. A study from china also found that parents’ command and reprimand were negatively correlated with adolescents’ self-esteem and emotional balance, and positively related to social anxiety and behavior problems (Fang et al., 2006). In addition, Krohne and Hock (1991) and Dumas et al. (1995) found that mother of daughter with high social anxiety was inclined to control and help her daughter in the daily life and study. Furthermore, father of children with high anxious showed more controlling behavior (Greco and Morris, 2002). Most of these studies, however, mainly focused on the children’s parenting styles and social anxiety in non-migrant families, while few studies examined the relationship between parenting styles and adolescents’ social anxiety in migrant families.

Thus, the objective of this study is to explore the relationship between adolescents’ social anxiety and parenting styles in migrant families. And the expected results are that positive parenting styles (e.g., emotional warmth) will be negatively correlated with adolescents’ social anxiety, and can reduce adolescents’ social anxiety; whereas negative parenting styles (e.g., rejection and overprotection) will be positively related to adolescents’ social anxiety and can increase their social anxiety. This study is crucial to provide scientific evidence for developing migrant family training and education program and to promote the holistic development of children in migrant families in the future.

## Materials and Methods

### Participants

A total of 1,345 students from four non-government-funded junior middle schools in Guangdong province in China formed the research sample. All the students followed their parents from rural areas to the cities for more than 6 months. The students were aged between 11 and 19 (Mean = 13.70, *SD* = 1.14), more than half of the participants were male students (59.26%). The majority of students lived with their parents (90.36%) and belonged to the Han ethnic group (92.80%); 52.87% of the students had one sibling and 32.59% of them had more than one sibling. These students came from all three grades of the junior middle schools, with 35.15% from Grade 1, 30.54% from Grade 2 and 34.31% from Grade 3, respectively.

Ethical approval for the study was granted by the Ethics Committee of National Research Institute for Family Planning, Beijing, China. All participants volunteered to participate in this study and signed statements of their informed consent. All subjects were informed that their participations were not paid.

### Procedure

The research was supported by the local government and education committees. The students enrolled in the four non-government-funded middle schools were mostly from migrant families. Permission for the study was granted by the school principals and teachers. The quantitative research method such as questionnaire survey was employed in this study. The questionnaire survey was conducted in the classroom by trained psychologists and graduate students with psychology major. The total time used for the survey was around 10 min.

### Instruments

#### Short-Form of the Egna Minnen Beträffande Uppfostran: One’s Memories of Upbringing (s-EMBU)

The Chinese version of the s-EMBU, which was revised by Li et al. (2012) according to the modification of the original short-Egna Minnen Beträffande Uppfostran (Arrindell et al., 1999), was used in this study. The revised Chinese version evaluates parental rearing behaviors in three dimensions using two versions, father version and mother version, with each including Rejection (six items), Emotional warmth (seven items), and Overprotection (eight items). Therefore, the Chinese version of s-EMBU consists of 21 items, with answers on a 4-point Likert scale (1: no, never; 2: yes, occasionally; 3: yes, often; 4: yes, always). The revised Chinese version had relatively high reliability, and the alpha coefficient was from 0.71 to 0.80 (Li et al., 2012) and test–retest reliability coefficient after 10 weeks was from 0.70 to 0.81 (Jiang et al., 2010), which illustrated a high internal consistency between father version and mother version. In this study, we computed the Cronbach’s Alpha coefficient and split-half reliability coefficient, ranging from 0.66 to 0.82 and from 0.65 to 0.82, respectively.

#### Social Anxiety Subscale of Self-Consciousness Scale

This scale was used to measure the subjective anxiety, the difficulty of language expression and behavior. It contains six items including unfamiliar places, being stared at, embarrassing incident, talking to strangers, public speaking, and a large crowd of people. Students’ responses to the items are scored by using a 4-point Likert scale (1: no, not like me, 2: yes, a bit like me, 3: yes, most of the time, 4: yes, very much like me), and higher scores indicates higher degree of anxiety. The modification of Chinese version was used in this study. The correlation coefficient between the revised scale and the original scale was 0.86 (Scalise and Carver, 1985). Using the present data, the Cronbach’s Alpha coefficient was 0.89.

### Statistical Analysis

Both the EpiData 3.1 and SPSS softwares were used for data entry, cleaning and statistical analysis. The statistical methods used here included descriptive analysis, *t*-test, ANOVA test, correlation analysis and regression analysis, etc. The descriptive analysis was used to describe the distributions of parenting styles under various demographic variables, and the differential analysis was conducted using *t*-test and ANOVA test. The social anxiety scores of all participants were sorted in a descending order first, and then the participants with scores in the top 25% and those in the bottom 25% were selected as the high social anxiety group and low social anxiety group, respectively. Group differences between different degrees of social anxiety for parenting styles were also analyzed. In addition, the relationship between parental rearing and social anxiety was investigated using correlation analysis and regression analysis.

## Results

### Descriptive Statistics and Variation Analysis

**Table 1** shows the descriptive statistics and inference statistics (ANOVA and *t*-test) regarding the adolescents-reported parenting styles and social anxiety under different demographic variables. The results indicated that there were no significant differences in social anxiety among different genders, grades and numbers of siblings, and no significant differences were observed in parenting styles among different children genders and numbers of siblings (*p* > 0.05). However, the significant differences in parents’ emotional warmth were found among different grades (*p* < 0.01); more specifically, the scores of parents’ emotional warmth gradually decreased as the grade increases. In addition, the paired *t*-test revealed that there were significant differences between father’s rearing styles and mother’s rearing styles in all three types of parenting styles (*p* < 0.01), and the scores of emotional warmth, overprotection and rejection from mothers were significantly higher than those from fathers.

**Table 1:** Descriptive statistics and inference statistics (ANOVA and *t*-test) regarding the parenting styles and social anxiety under different demographic variables.

		Father’s rejection	Mother’s rejection	Father’s emotional warmth	Mother’s emotional warmth	Father’s overprotection	Mother’s overprotection	Social anxiety
Gender	Boys	1.69 ± 0.60	1.68 ± 0.58	2.25 ± 0.67	2.35 ± 0.67	2.06 ± 0.48	2.15 ± 0.48	2.35 ± 0.62
	Girls	1.63 ± 0.59	1.74 ± 0.64	2.23 ± 0.69	2.31 ± 0.69	2.04 ± 0.51	2.19 ± 0.55	2.34 ± 0.62
	*t*-value	1.64	-1.61	0.44	0.84	0.86	-1.30	0.11
Grade	Junior one	1.67 ± 0.60	1.68 ± 0.61	2.33 ± 0.66	2.43 ± 0.65	2.03 ± 0.47	2.16 ± 0.49	2.35 ± 0.60
	Junior two	1.67 ± 0.62	1.73 ± 0.64	2.22 ± 0.72	2.31 ± 0.69	2.04 ± 0.51	2.15 ± 0.49	2.34 ± 0.59
	Junior three	1.65 ± 0.58	1.70 ± 0.56	2.17 ± 0.65	2.25 ± 0.68	2.08 ± 0.49	2.19 ± 0.53	2.35 ± 0.65
	*F*-value	0.65	0.13	6.68^∗∗^	7.78^∗∗∗^	1.14	0.78	0.07
Number of Siblings	Single-child	1.67 ± 0.63	1.68 ± 0.59	2.31 ± 0.67	2.41 ± 0.67	2.09 ± 0.49	2.23 ± 0.49	2.31 ± 0.64
	One sibling	1.67 ± 0.58	1.70 ± 0.59	2.24 ± 0.69	2.33 ± 0.68	2.05 ± 0.49	2.15 ± 0.51	2.34 ± 0.61
	More siblings	1.67 ± 0.61	1.72 ± 0.64	2.18 ± 0.66	2.29 ± 0.67	2.04 ± 0.49	2.15 ± 0.52	2.39 ± 0.62
	*F*-value	0.01	0.32	2.43	2.02	0.69	1.72	1.41
Total		1.66 ± 0.59	1.70 ± 0.60	2.23 ± 0.68	2.32 ± 0.67	2.05 ± 0.49	2.16 ± 0.51	2.35 ± 0.62
	*t-*value	-3.28^∗∗^		-8.44^∗∗∗^		-12.62^∗∗∗^		


*^∗∗^*p* < 0.01, ^∗∗∗^*p* < 0.001.*

### Differences between Different Groups of Social Anxiety on Parenting Styles

As can be seen from **Table 1**, the mean score of social anxiety was 2.35 ± 0.62. According to the 25% classification criteria, all the participants were divided into two groups: high anxiety group and low anxiety group (see **Table 2**). The results from independent-samples *t*-tests indicated that significant group differences between two anxiety groups were present in both father’s rearing styles and mother’s rearing styles. To be specific, more parental emotional warmth was reported in the low social anxiety group than that in the high social anxiety group; moreover, the mean scores of parental rejection and overprotection were significantly higher the in high social anxiety group than those in the low social anxiety group.

**Table 2:** Differences between different groups of social anxiety on parenting styles.

	High social anxiety (Mean ± *SD*)	Low social anxiety (Mean ± *SD*)	*t*-value
**Father**			
Rejection	1.79 ± 0.65	1.62 ± 0.65	3.45^∗∗∗^
Emotional warmth	2.14 ± 0.61	2.34 ± 0.74	-3.74^∗∗^
Overprotection	2.13 ± 0.51	2.00 ± 0.51	3.25^∗∗^
**Mother**			
Rejection	1.83 ± 0.65	1.64 ± 0.60	3.86^∗∗∗^
Emotional warmth	2.26 ± 0.62	2.41 ± 0.74	-2.74^∗^
Overprotection	2.25 ± 0.53	2.11 ± 0.53	3.21^∗∗^


*^∗^*p* < 0.05, ^∗∗^*p* < 0.01, ^∗∗∗^*p* < 0.001.*

### Associations between Parenting Styles and Adolescents’ Social Anxiety in Migrant Families

#### Correlations between Parental Rearing Patterns and Social Anxiety

**Table 3** provides the correlations between adolescent-reported parental rearing patterns and social anxiety. The results showed that the adolescent-reported parenting styles were related to adolescents’ social anxiety. Specifically, both parents’ rejection and overprotection were positively correlated with adolescents’ social anxiety, and father and mother’s emotional warmth were negatively associated with the social anxiety of adolescents. The degrees of adolescents’ social anxiety in migrant families were lower when they perceived more emotional warmth from their father or mother. The results also indicated that father’s rejection was negatively associated with parents’ emotional warmth, and positively correlated with parents’ overprotection and mother’s rejection. However, there were no significant relationships between father’s emotional warmth and parental overprotection, as well as between mother’s emotional warmth and parental overprotection.

**Table 3:** Correlations among father’s rearing pattern, mother’s rearing pattern, and social anxiety.

	Rejection (father)	Emotional warmth (father)	Overprotection (father)	Rejection (mother)	Emotional warmth (mother)	Overprotection (mother)	Social anxiety
Rejection (father)	1						
Emotional warmth (father)	-0.32**	1					
Overprotection (father)	0.49**	0.04	1				
Rejection (mother)	0.74**	-0.25**	0.45**	1			
Emotional warmth (mother)	-0.25**	0.83**	-0.00	-0.35**	1		
Overprotection (mother)	0.45**	0.01	0.80**	0.54**	-0.01	1	
Social anxiety	0.13**	-0.14**	0.12**	0.13**	-0.09**	0.12**	1


*^∗∗^*p* < 0.01.*

#### Regression Analysis

Stepwise regression analysis was conducted with social anxiety as a dependent variable and adolescent-reported parenting styles as independent variables (see **Table 4**). The results illustrated that father’s emotional warmth entered the regression model first, followed by mother’s overprotection. Father’s emotional warmth could explain 2% of variation of the social anxiety of the adolescents from migrant families (i.e., *R*^2^ = 0.020, Adjust *R*^2^ = 0.019) in model 1. Both father’s emotional warmth and mother’s overprotection could explain 3.5% of variance in social anxiety (i.e., *R*^2^ = 0.035, Adjust *R*^2^ = 0.033) in model 2. Father’s emotional warmth was negatively correlated with adolescents’ social anxiety, while mother’s overprotection was positively correlated with adolescents’ social anxiety.

**Table 4:** The stepwise regression models^a^.

	Model	*R*^2^	Adjust *R*^2^	Unstandardized coefficients		Standardized coefficients	*t*	Sig.
							
				*B*	Standard error	Beta	
1		0.020	0.019				
	(Constant)			9.750	0.361		26.99	0.000
	Emotional warmth (father)			-0.757	0.154	-0.140	-4.90	0.000
2		0.035	0.033				
	(Constant)			7.845	0.565		13.88	0.000
	Emotional warmth (father)			-0.762	0.153	-0.141	-4.97	0.000
	Overprotection (mother)			0.886	0.203	0.123	4.36	0.000


*^a^Dependent variable: social anxiety. Independent variables: rejection (father, mother); emotional warmth (father, mother); overprotection (father, mother).*

## Discussion

This study investigates the relationship between adolescent-reported parenting styles and adolescents’ social anxiety in migrant families. The result suggests that similar parenting profiles for father and mother are perceived in all three parenting dimensions (rejection, emotion warmth, and overprotection); the mean scores of parents’ emotional warmth are the highest, followed by the mean scores of overprotection, and the mean scores of rejection is the lowest. Emotional warmth and other positive behaviors are the main characteristics of parental rearing behaviors, which confirms the findings of other Chinese scholar’s studies (Fang et al., 2006). This result indicates that the adolescents in migrant families and non-migrant families exemplify similar patterns of parenting styles (Liu et al., 2015). Both the parents of the children in migrant families and non-migrant families all wanted to take care of their children using warm and caring ways (Xu et al., 2015). The current study also find that there are significant differences between mother’s parenting styles and father’s parenting styles, and the mean scores of mother’s parenting styles are higher than those of father’s. This indicates that parents play different roles in children rearing and mothers are more involved in caretaking and comforting (Paquette, 2004). Especially for the migrant families, mothers often spend more time at home, whereas fathers have to go out to work because they are the main providers of family income. Thus, mothers may play more important role than fathers in raising children (Forehand and Nousiainen, 1993; Paulson and Sputa, 1996; Castro et al., 1997). Generally, Chinese mothers always do more in bringing up and educating their children. This is the embodiment of traditional Chinese family education model, just like a Chinese saying, ‘a mother always worries about her traveling child.’ Children and adolescents usually ask mothers for help when they have a variety of needs and requirements.

The results of this study show that social anxiety for adolescents in migrant families are positively correlated with parental rejection and overprotection, and negatively correlated with parental emotional warmth. Evidence also indicates that parents’ negative rearing behaviors such as overprotection, rejection, punishment, and less emotional warmth are related to childhood anxiety (Bögels and Phares, 2008; Degnan et al., 2010). For the adolescents’ parents in migrant families, they may be more blind and subjective, and they are more likely to incline to use punishment and harsh parenting when educating children, because most of them have lower socioeconomic status and level of education (McLoyd, 2008). McLoyd (2008) had pointed out that the parents who had lower socioeconomic status had not enough time and energy to meet the needs of their children’s attachment, resulting in emotional neglect. The results of this study also suggest that parents of children with high anxious are likely to be less accepting and more overprotective than those of low anxious children, which is consistent with previous studies (Bögels et al., 2001; Festa and Ginsburg, 2011). When adolescents in migrant families feel more emotional warmth from their parents, their social anxiety level may be reduced. Conversely, the lacking of parental care, understanding and emotional warmth will result in distrust and a sense of insecurity, which can undermine their social interaction.

In addition, the results of this study also find that in migrant families, fathers’ emotional warmth can reduce adolescents’ social anxiety, whereas maternal overprotection can increase it. Moreover, father’s emotional warmth has the strongest association with the low social anxiety for adolescents in migrant families. This result is in line with the studies which explore the father’s role in children’s social anxiety (Greco and Morris, 2002). Also, some evidences found that lack of fathers’ emotional warmth had a relatively stronger effect for patients with social anxiety disorder compared to mothers (Arrindell et al., 1983). In addition, the results from this study support that fathers play a moderately more important role in the adolescent’s social development than mothers, and that they may have a stronger influence in the development of social anxiety in particular (Bögels and Phares, 2008; Majdandžić et al., 2010). Especially for Chinese migrant families, fathers, in general, should be the main providers of family income, and therefore they have to spend more time and energy on work to cope with job mobility and instability, leaving them little time and opportunity to interact with their children or express their understanding and emotional warmth. However, a father often has a higher status in a traditional Chinese family, being regarded as the head of the family, to be respected and obeyed by all family members. Therefore, father’s emotional warmth may be more important to adolescents’ social development, compared to their mother’s emotional warmth.

This study also indicates that mother’s overprotection is associated with adolescents’ social anxiety in migrant families. More specifically, parents’ overprotective attitudes, especially mothers’ attitudes, can increase children’s social anxiety (Verhoeven et al., 2012), and maternal overprotection is related to boy’s general anxiety (Rorka and Morrisa, 2009). Hock and Krohne (2004) found that mothers of children with higher social anxiety displayed more overprotective and controlling behaviors. In China, the traditional rearing mode of “stern father and compassionate mother” still prevails in most families. Mothers often adopt a protective stance toward their children, so as to conform to the image of “compassionate mother.” However, many previous studies have revealed that maternal behavior might be strongly related to children’s anxiety (e.g., Verhoeven et al., 2012). Although mothers’ protection and care can help children deal with problems and setbacks, their overprotection can lead to the increase of their children’s attachment anxiety levels; and too much denial and protection from mothers can result in children’s non-confidence and dependence (Ma and Wang, 2015). Moreover, overprotected children often have difficulty interacting with the outside world, are unable to build good social skills, and show social withdrawal in interpersonal communication, thereby causing their social anxiety levels increase. Therefore, parental overprotection, especially mothers’ overprotection, may prevent the development of individual independence and social skills. It follows that parental rearing patterns, attitudes and behaviors may have an important impact on children’s psychology, behavior and social development.

### Implications for Services

The results from this study suggest that the parental rearing pattern indeed has an effect on social anxiety for adolescents in Chinese migrant families. This promotes a better understanding of the relationship between parenting styles and social anxiety. A possible prediction is that parents who are warmer, less rejecting and less likely to resort to punishment may bring up more sociable and emotionally stable children, yet more rigorous evidence is welcome in future studies. In addition, people should show more concern for the adolescents’ social anxiety, and improve the patterns of adolescents’ rearing in migrant families. Social resources should be provided to help parents in migrant families receive more family education and parental rearing training to benefit the adolescents’ physical and psychological development. We firmly believe that to improve children’s mental health in migrant families, it is very crucial to improve their parents’ rearing styles and cultivate the positive social interaction behavior.

### Limitations and Future Directions

The limitations of this study include the lack of comparison of parenting styles and anxiety of adolescents between in migrant families and non-migrant families. Only self-report measures from adolescents themselves in this study may have subjective bias, thus data from multiple reporters such as children, parents, and teachers should be collected in further studies. And the birth order is not considered in the analysis of demographic statistics, thus the differences in children’s social anxiety between different birth orders under the same rearing mode, and the differences in social anxiety between single-child and the first-born of more children under different rearing modes should be further investigated in the future. Last, but not least, another line of research worth considering is to explore the interaction of gene and environment because anxiety may have a heritable component.

## Author Contributions

JX: Substantial contributions to the design of the work, data analysis and writing. SN: Substantial contributions to the design of the work, data collection. MR: Substantial contributions to revising the work critically. CZ: Substantial contributions to acquisition of data for the work.

## Conflict of Interest Statement

The authors declare that the research was conducted in the absence of any commercial or financial relationships that could be construed as a potential conflict of interest.
